# Optimization of Peri-Implant Bone Repair in Estrogen-Deficient Rats on a Cafeteria Diet: The Combined Effects of Systemic Risedronate and Genistein-Functionalized Implants

**DOI:** 10.3390/ma18030662

**Published:** 2025-02-02

**Authors:** Tatiany Aparecida de Castro, Jaqueline Suemi Hassumi, Gabriela Morais Julião, Marina Corrêa Dutra, Ana Cláudia Ervolino da Silva, Naara Gabriela Monteiro, Fábio Roberto de Souza Batista, Gabriel Mulinari-Santos, Paulo Noronha Lisboa-Filho, Roberta Okamoto

**Affiliations:** 1 Departamento de Ciências Básicas, Faculdade de Odontologia de Araçatuba, Universidade Estadual Paulista Júlio de Mesquita Filho, UNESP, Araçatuba 16015-050, SP, Brazil; tatiany.castro@unesp.br (T.A.d.C.); jaqueline.hassumi@unesp.br (J.S.H.); g.juliao@unesp.br (G.M.J.); marina.dutra@unesp.br (M.C.D.); ana.ervolino@unesp.br (A.C.E.d.S.); naara.monteiro@unesp.br (N.G.M.); fabio.rs.batista@unesp.br (F.R.d.S.B.); 2 Departamento de Física e Meteorologia, Faculdade de Ciências de Bauru, Universidade Estadual Paulista Júlio de Mesquita Filho, UNESP, Bauru 17033-360, SP, Brazil; paulo.lisboa@unesp.br

**Keywords:** osteoporosis, osseointegration, genistein, layer-by-layer, nanoparticles

## Abstract

Estrogen deficiency, coupled with a cafeteria diet (CD), can impair peri-implant bone repair, posing a significant challenge to implant success in affected individuals. Thus, it is crucial to explore strategies for implant functionalization and systemic treatments that could alleviate these bone alterations. This study aimed to assess peri-implant bone repair in ovariectomized (OVX) rats subjected to a CD, with a focus on implants functionalized with genistein (GEN), compared to conventional implants (CONV), and the effects of systemic treatment with risedronate sodium (RIS). In total, thirty-six female rats were assigned to three groups: rats with estrogen (SHAM), rats with estrogen deficiency and CD (OVX-CD), rats with estrogen deficiency, CD, and systemic RIS treatment (OVX-CD-RIS). All rats underwent bilateral extraction of the first upper molars followed by implant installation. Each group was further subdivided based on implant type: conventional implants (CONV) or GEN-functionalized implants, resulting in six subgroups (*n* = 6). The study employed several analyses, including reverse torque testing, microcomputed tomography (Micro-CT), epifluorescence microscopy, and molecular assays. The main result demonstrated that the OVX-CD-RIS/GEN subgroup exhibited significantly higher reverse torque values, indicating stronger implant stability. Micro-CT scans revealed a greater bone volume in the OVX-CD-RIS/GEN subgroup compared to other subgroups. Epifluorescence microscopy also demonstrated an increased mineral apposition rate in both the OVX-CD/GEN and OVX-CD-RIS/GEN subgroups. Molecular analysis indicated elevated expression levels of osteocalcin, alkaline phosphatase, and vascular endothelial growth factor in the OVX-CD-RIS/GEN subgroup. In conclusion, the combined treatment of systemic RIS and GEN-functionalized implants significantly enhanced peri-implant bone repair, offering a promising strategy to improve implant outcomes in postmenopausal women with metabolic syndrome.

## 1. Introduction

Osteoporosis has become increasingly prevalent worldwide due to rising life expectancy [1,2]. There are roughly 10 million people with osteoporosis just in the United States [3]. Especially, postmenopausal women face a higher risk of osteoporosis and fractures, primarily due to estrogen deficiency [4]. This condition poses a significant public health challenge, affecting one in three women aged over 50 years [4,5,6]. Additionally, studies have linked metabolic syndrome to postmenopausal women [7], driven principally by a cafeteria diet (CD) with high levels of fat, sugar, and sodium [8]. Recently, the global prevalence of metabolic syndrome is estimated to range from 7% to 46% [7,8,9,10]. Osteoporosis and metabolic syndrome contribute to catabolic changes in bone [7,11], highlighting the need for effective therapeutic strategies to maintain peri-implant bone repair under these compromised conditions.

Risedronate (RIS), a third-generation bisphosphonate, is widely prescribed for the treatment of osteoporosis, particularly among postmenopausal women [12,13]. Clinical trials have demonstrated that RIS reduces fracture risk and improves bone density in this population [7,14]. RIS has a modified molecular structure, enhancing its efficacy while minimizing side effects, particularly regarding gastrointestinal complications [7,14]. RIS is an antiresorptive agent that primarily reduces osteoclast activity [15], and therefore can increase alveolar bone density [16]. Notably, there are concerns about the risk of osteonecrosis of the jaw associated with bisphosphonates [7,11,12,14]. However, RIS has demonstrated a lower risk of this complication, since it is administered orally rather than intravenously [17,18]. Consequently, RIS may offer a promising strategy for supporting peri-implant bone repair, particularly in catabolic conditions.

Genistein (GEN) is a natural isoflavonoid classified as a phytoestrogen, acting as a selective modulator of estrogen receptors [19]. It is widely used in the prevention and treatment of postmenopausal bone loss, as it stimulates the expression of estrogen receptor α, thereby promoting bone formation through osteoblast differentiation and enhancing vascularization [13,20]. Previous studies have shown that GEN is not cytotoxic to MC3T3-E1 cells and osteoblasts [13,20]. Additionally, systemic administration of GEN in animal models with estrogen deficiency has shown positive effects on alveolar and peri-implant bone repair [20]. Prior in vitro studies have indicated that GEN promotes mineralization in osteoblast cultures [21]. Thus, genistein, particularly when used in functionalized form, has the potential to mitigate the antiresorptive effects associated with estrogen deficiency and CD.

This study employed ovariectomized (OVX) rats to simulate estrogen deficiency, alongside a CD to mimic the inadequate dietary habits of metabolic syndrome; thus, these conditions are similar to those experienced by postmenopausal women [7]. The CD is considered an effective condition for inducing metabolic syndrome in rats [8,9,16]. Specifically, this study investigates the potential of systemic RIS treatment and GEN-functionalized implants to improve bone repair in this compromised model. By combining RIS and GEN-functionalized implants, this approach targets both the prevention of bone resorption via RIS and the promotion of bone formation and vascularization via GEN. This dual mechanism optimizes peri-implant bone repair, addressing the limitations of single treatments. Few studies have explored combining systemic and local therapies for enhanced peri-implant healing. Consequently, combination therapy may significantly improve bone repair outcomes, especially in compromised conditions, such as estrogen deficiency and metabolic syndrome. The peri-implant bone repair was evaluated through biomechanical, microcomputed tomography (Micro-CT), epifluorescence, and molecular analyses in OVX rats subjected to a CD, experiencing both estrogen deficiency and CD while receiving the combined treatment with systemic RIS and local GEN-functionalized implants.

## 2. Materials and Methods

### 2.1. Ethical and Study Design

After the study obtained approval from the Ethics Committee under protocol number 566–2019 and according to the ARRIVE guideline [22]. Initially, thirty-six female Wistar rats (four months old and weighing around 350 g; *Rattus novergicus albinus*) were randomly divided into three groups: rats with estrogen (SHAM), rats with estrogen deficiency and CD (OVX-CD), rats with estrogen deficiency, CD, and systemic treatment with RIS (OVX-CD-RIS). After bilateral extraction of the first upper molars, implants were installed immediately. Then, each group was subdivided into two subgroups based on implant type placed (*n* = 6): CONV implants or GEN-functionalized implants. The six subgroups are presented in Table 1.

Following OVX surgeries, the animals of OVX-CD and OVX-CD-RIS groups received CD. After 30 days of OVX surgeries, the rats in the OVX-CD-RIS group started treatment with RIS via oral gavage. After 90 days of OVX surgeries, the first upper molars were extracted bilaterally and immediately installed were implants of the same type.

### 2.2. Estrous Cycle

The estrous cycle was checked to ensure that the rats selected for the experiments had normal cycles. They were placed in individual cages and between 1 and 2 drops of saline solution were introduced daily into the vagina, which were then collected and placed on a histological slide for reading. Immediate microscopic examination was conducted to recognize the 4 phases of the estrous cycle. The rats were used after obtaining 2 to 3 regular estrous cycles. It is worth noting that this technique was used again to validate the bilateral ovariectomy surgery performed [23].

### 2.3. OVX Surgeries

The OVX surgeries were performed on the animals fasted for eight hours. Thus, the rats in the OVX-CD and OVX-CD-RIS groups were anesthetized with 100 mg/kg of ketamine (Vetaset—Fort Dodge Saúde Animal Ltda., Campinas, São Paulo, Brazil) intramuscularly and 5 mg/kg of xylazine (Dopaser—Laboratório Calier do Brasil Ltd.a.—Osasco, São Paulo, Brazil) intraperitoneally. The SHAM group underwent the same procedure, but only the surgical exposure of the uterine horns and ovaries was performed without removal, in order to subject the animals to the same surgical stress. The OVX surgery followed a previously established technique [24].

### 2.4. CD Diet

Subsequent to OVX surgery, the animals in the OVX-CD and OVX-CD-RIS groups were placed on a CD diet. These animals received a high-fat diet to mimic the unhealthy habit of a metabolic syndrome condition. For each animal, a 30 g portion of unhealthy food products was weighed daily. The rats were fed with the following: Stuffed Cracker 10 g (Pandurata Alimentos Ltd.a, Guarulhos, São Paulo, Brazil), Wafer 10 g (Pandurata Alimentos Ltd.a, Guarulhos, São Paulo, Brazil), Corn Chips 10 g (Indústria e comércio de biscoitos e salgados Keleck Ltd.a, Jales, São Paulo, Brazil). A bottle of water with a 12% sucrose concentration was also available, with water + sucrose (12% solution): 24 g of sucrose (Camil Alimentos S.A., São Paulo, São Paulo, Brazil) + 200 mL of water per day. The CD diet was prepared following prior models [8,24,25]. The other animals without CD were fed with a balanced diet (1.4% Ca and 0.8% Pe, Nuvilab, Curitiba, PR, Brazil).

### 2.5. RIS Administration

After 30 days of post-OVX, the rats in the OVX-CD-RIS/CONV and OVX-CD-RIS/GEN subgroups started systemic treatment with RIS (2-3-pyridinyl-1-hydroxyethylidene-bisphosphonic acid; ApothiMed Ltd.a., Araçatuba, SP, Brazil) via oral gavage at a dosage of 0.7 mg/kg/weekly. It was kept until euthanasia.

The other subgroups were administered only vehicles of physiological solution 0.9% (Fisiológico^®^, Laboratórios Biosintética Ltd.a., Ribeirão Preto, SP, Brazil).

These administrations were performed with a curved gavage needle with the medication diluted in potable water. The gavage needle was used to bring the medication close to the esophagus region and the final volume administered was 0.3 mL for all rats. These steps were followed according to a prior study [25].

### 2.6. Implants Functionalized with GEN

Commercially pure grade IV titanium implants based on the double acid attack concept (Medens, Itu, São Paulo, Brazil) were used, with a diameter of 1.4 mm and a height of 2.7 mm, sterilized by gamma rays. The implants of the GEN subgroups were functionalized using the layer-by-layer technique. In the functionalization, the implants were submerged in sulfonated polystyrene electrolyte with a negative charge for 5 min and washed with stock water. Afterwards, implants were immersed in a TiO_2_ electrolyte solution with a positive charge, previously prepared by the sol–gel method for 5 min, washed with stock water, and, finally, the implants were submerged in a concentration of 100 µg of GEN for 10 min and then washed in Milli-Q water (Millipore, Burlington, MA, USA). Our previous in vitro study in Patents showed that this concentration of GEN ensures cell viability and non-genotoxicity. Specifically, the technique for functionalization with GEN was also included in Patents deposited and protected by patent number BR 10 2021 019134 1 from Brazil.

### 2.7. Dental Extraction and Implant Placement

After 90 days of OVX surgery, the first upper molar extraction and immediate implant installation bilaterally were performed in all groups. The technique used for tooth extraction and implant installation was described previously [25]. The animals were fasted for eight hours before the surgery, sedated by the combination of 50 mg/kg intramuscular ketamine (Vetaset-Fort Dodge Saúde Animal Ltd.a., Campinas, SP, Brazil) and 5 mg/kg xylazine hydrochloride (Dopaser-Laboratório Calier do Brasil Ltd.a., Osasco, SP, Brazil), and received mepivacaine hydrochloride (0.3 mL/kg, Scandicaine 2% with adrenaline 1:100,000, Septodont, SaintMaur-des-Fossés, France) as local anesthesia. After sedation, the animal was positioned on a surgical table designed for rodents, favoring the maintenance of the oral cavity opening, and providing adequate positioning for extraction. After antisepsis with topical polyvinylpyrrolidone iodine (PVPI 10%, Riodeine Degermante, Rioquímica, São José do Rio Preto, SP, Brazil) was performed, the extraction of the upper first molar was executed with the aid of a Hollenbeck 3S carver (Quinelato, Rio Claro, SP, Brazil).

Immediately after the tooth extraction, bilaterally, the implants were installed in the maxilla. The implants were pure grade IV titanium with double acid etching (diameter of 1.4 mm and a height of 2.7 mm; Medens, Itu, São Paulo, Brazil). For this, a drilling was performed with a 1.2 mm diameter twist drill mounted on an electric motor (BLM 600^®^; Driller, São Paulo, SP, Brazil) at a speed of 1000 rpm under irrigation with 0.9% isotonic sodium chloride solution (Fisiológico^®^, Laboratórios Biosintética Ltd.a., Ribeirão Preto, SP, Brazil) and a contra-angle with a 20:1 reduction (Angle piece 3624N 1:4, Head 67RIC 1:4, KaVo^®^, Kaltenbach & Voigt GmbH & Co., Biberach, Baden-Württemberg, Germany). Each animal received two implants of the same type, CONV implants or GEN-functionalized implants, in the alveolar bone of the extracted upper first molar. All implants were inserted by a calibrated, blinded, and trained surgeon F.B.R-S. Suture was performed with monofilament thread (Nylon 5.0, Ethicon, Johnson, São José dos Campos, SP Brazil). In the immediate postoperative, animals received a single intramuscular dose of 0.2 mL of penicillin G-benzathine (Small Veterinary Pentabiotic, Fort Dodge Saúde Animal Ltd.a., Campinas, SP, Brazil).

### 2.8. Euthanasia

Euthanasia was performed 28 days after the implant surgeries. The euthanasia was performed with an intraperitoneal overdose of sodium thiopental (2.5%; 150 mg/kg; Fort Dodge Saúde Animal Ltd.a., Campinas, Brazil) and lidocaine (2%;10 mg/kg; Anesthetic, Laboratório Bravet Ltd.a., Rio de Janeiro, Brazil). The samples were collected for analysis.

### 2.9. Statistical Analysis

Biomechanical, Micro-CT, epifluorescence, and molecular analyses were performed. Statistical analysis was carried out with a significance level of 5%. Two-way ANOVA statistical analysis was performed with a significance level of 5%, where the factors considered were as follows: implant functionalization (GEN or CONV) and systemic condition (SHAM, OVX/CD, OVX/CD/RIS). Differences between subgroups were identified using the Tukey post-test (*p* < 0.05). The sample size was determined from the sample calculation by a power test performed at http://www.openepi.com/SampleSize/SSMean.htm (OpenEpi, version 3, open-source calculator; accessed on 7 March 2023). It obtained the mean number (*n*) of six animals per subgroup using the reverse torque data from a prior study [25].

### 2.10. Biomechanical Analysis (Reverse Torque)

Half of the collected samples were subjected to reverse torque analysis. For that, counterclockwise movement using an implant key adapted in the implant hexagon was performed in a digital torque wrench (TQ-680 Model, Instrutherm, São Paulo, Brazil). A counterclockwise movement was applied by increasing the reverse torque until the implant rotated within the bone, and completely ruptured the bone and implant interface, at which point the torque meter registered the maximum torque peak for this rupture in Newton centimeters (Ncm). The reverse torque was according to a prior study [25].

### 2.11. Micro-CT

The other half of the samples were submitted to Micro-CT followed by epifluorescence analysis. For Micro-CT analysis, samples were reduced and stored in 70% alcohol. Firstly, they were subjected to analysis by X-ray beam scanning in a Micro-CT system (SkyScan 1272 Bruker MicroCT, Aatselaar, Belgium). The pieces were scanned by the SkyScan microtomography machine using 9 µm thick sections, with a Copper and Aluminum filter and a rotation step of 0.3 mm. The images obtained from the samples were stored and reconstructed by determining the area of interest in NRecon software (SkyScan, 2011; Version 1.6.6.0). In Data Viewer software (SkyScan, Version 1.4.4 64-bit), the images were reconstructed and adapted to standard positioning and could be observed in three planes: transverse, longitudinal, and sagittal planes. Using the CTAnalyser—CTAn software (SkyScan CTAn, v1.12.4.0; Leuven, Belgium), a rectangular area around the implant (ROI) delimited by 0.5 mm in width and 0.8 mm in height was defined around the entire implant. This ROI was defined to calculate the bone volume (BV, mm^2^). The software analyzed and measured the image according to gray scales in threshold. The threshold used in the analysis was 25–90 shades of gray, which made it possible to obtain the bone volume around the implants. The Micro-CT followed the previous guideline [26].

### 2.12. Epifluorescence

The same samples that were scanned in the Micro-CT were subjected to confocal microscopy for epifluorescence analysis. It is worth noting that the fluorochromes to be evaluated were injected at 14 days (calcein, 20 mg/kg) and 24 days (alizarin, 30 mg/kg), being euthanized at 28 days postoperatively. Therefore, maxillae were removed and fixed in a 10% formaldehyde solution for 48 h, washed in running water for 24 h, and kept in alcohol 70 for microtomographic evaluation. At the end of this analysis, they went through the dehydration process. After dehydration, these pieces were immersed in a 100-alcohol solution and thermo-polymerizable resin gradually in different concentrations until we only used the resin as a form of immersion. For cuts and wear, the Exakt Cutting System automatic cutting and polishing system was used, until we obtained a section of approximately 80 μm thick from the pieces we used to make the blades. The analysis was carried out using a Leica CTR 4000 CS SPE (Leica Microsystems, Heidelberg, Germany) confocal laser microscope. The images obtained in different sections in the region around the implants were compressed to obtain the best fit. Once the thickness was selected, all sections were obtained and operated using the z-stack to better adjust the images that represented the best section of each animal in the experimental groups. These images are 1 × 1 mm^2^ in size and correspond to optical sections measuring 512 by 512 pixels. Sections of 2 μm were scanned for 2.5 min. Thus, 28 slices were obtained for every 56 μm of scan. BP 530/30 nm and 590 LP barrier filters were used, combined with double dichroic activation 488/568 nm, and the photomultiplier was set at 534 for calcein and 357 for alizarin. The codes 534 and 357 nm represented the filters that allowed the visualization of fluorochromes. One of the filters allowed the visualization of calcein in a blue filter and the other of alizarin in a green filter. These images were then reconstructed through the software stack that is installed to manipulate the Leica CTR 4000 CS SPE confocal microscope. The region around the implants presents two overlays of fluorochromes (calcein and alizarin) and each overlay represents an overlay of calcium that was precipitated in each of the periods showing the conversion of old bone to new bone. These images were saved in TIFF format and transported to ImageJ software (version 1.52v, National Institutes of Health, Bethesda, MD, USA). Using the color threshold tool, images were standardized according to hue, saturation, and brightness to primarily reveal fluorochromes. First, the calcein was highlighted, and the measuring tool was used to provide the area in μm^2^. The same was performed for alizarin, obtaining data on the dynamics of the alveolar bone. The first fluorochrome administered was calcein (green), which thus represents an old bone. After 10 days of applying calcein, an injection of alizarin was performed. Therefore, the bone marked with alizarin (red) fluorochromes represents newly mineralized bone, highlighting areas of active calcification. In this way, by quantifying the distance between the fluorochrome ranges, and relating it to the difference in 10 days between each injection, it was possible to calculate the daily mineral deposition rate (MAR), in accordance with a previous study [25].

### 2.13. Molecular Analysis

The samples from reverse torque were immediately removed with the aid of a gouge tool, thus collecting the peri-implant bone. Real-time polymerase chain reaction (PCR) was performed to evaluate the gene expression of markers related to bone repair. Each bone fragment was carefully washed in PBS and subsequently frozen in liquid nitrogen so that the total RNA could be extracted with Trizol reagent (Life Technologies: Invitrogen, Carlsbad, CA, USA). After analyzing the integrity, purity, and concentration of the RNA, cDNA was prepared using 1 µg of RNA through the reverse transcriptase reaction (M-MLV reverse transcriptase: Promega Corporation, Madison, WI, USA). The cDNAs of the samples were pipetted together with Taqman Fast Advanced Mastermix (Applied Biosystems, Foster City, CA, USA) in the PCR plate (96 well fast thermal cycling, Life Biotechnologies, Carlsbad, CA, USA) for detection of genes involved in the bone repair process (Taqman Gene Expression Assays, Carlsbad, CA, USA). Real-time PCR was performed in the Step One Plus real-time PCR detection system (Applied Biosystems) under the following conditions: 50 °C (2 min), 95 °C (10 min), and 40 cycles of 95 °C (15 s), 60 °C (1 min), followed by the standard denaturation curve. Relative gene expression was calculated about the expression of mitochondrial ribosomal proteins and normalized by the gene expression of the bone fragments undergoing repair from the different experimental periods (ΔΔCT method). The assay was performed in quadruplicate with ß-actin (Actb; Rn00667869_m1) as an endogenous control, according to an earlier study [24].

The RT-PCR was performed to assess the expression of osteocalcin (OC; BGGLAP; Rn00566386_g1), bone sialoprotein (IBSP; IBSP; Rn00561414_m1), tartrate-resistant acid phosphatase 5 (TRAP; Acp5; Rn00692676_g1), alkaline phosphatase (ALP; Alppl2; Rn01772432_g1), and vascular endothelial growth factor (VEGF; Vegfa; Rn01511602_m1), which were selected as markers to assess peri-implant bone repair. OC is a key marker of osteoblast activity and bone mineralization, reflecting osteoblastic differentiation during bone remodeling [27]. ALP, indicative of early osteoblast differentiation and mineralization, plays a critical role in active bone formation [27]. VEGF, essential for angiogenesis, supports vascularization in bone repair, which is crucial for osseointegration [28]. The corresponding gene sequences and primers used for the assays are provided in Table 2.

## 3. Results

### 3.1. Biomechanical Analysis (Reverse Torque)

**Figure 1:**
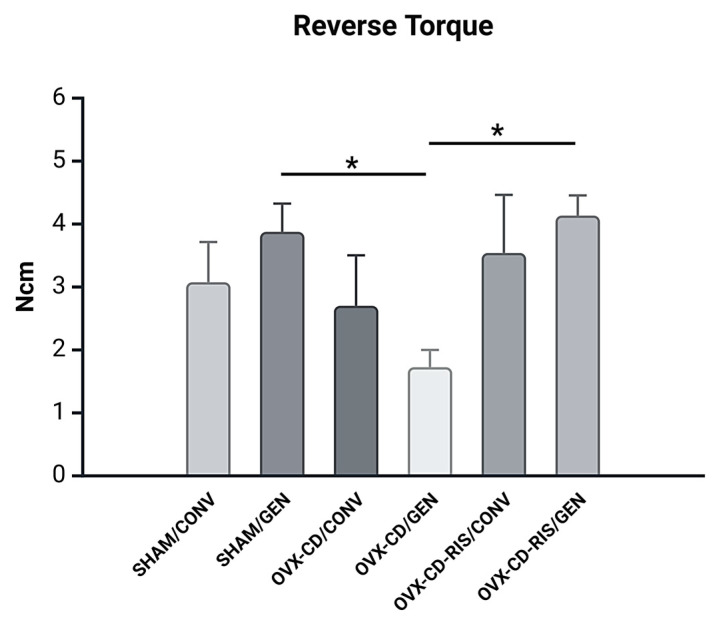
The removal torque values observed in the experimental groups were analyzed using a two-way ANOVA, with the systemic condition of the animals and the implant surface type as the factors of interest. If significant differences were detected between the factors, a Tukey post-test was applied to further investigate the results, with a significance level set at *p* < 0.05. An asterisk (*) denotes a statistically significant difference when compared to the OVX-CD/GEN subgroup.

The results showed that the OVX-CD-RIS/GEN subgroup obtained higher values and the OVX-CD/GEN obtained the lowest removal torque value (Figure 1). Statistically significant differences were observed between SHAM/GEN and OVX-CD/GEN (*p* = 0.0003) and between OVX-CD/GEN and OVX-CD-RIS GEN (*p* = 0.0002).

### 3.2. Micro-CT

**Figure 2:**
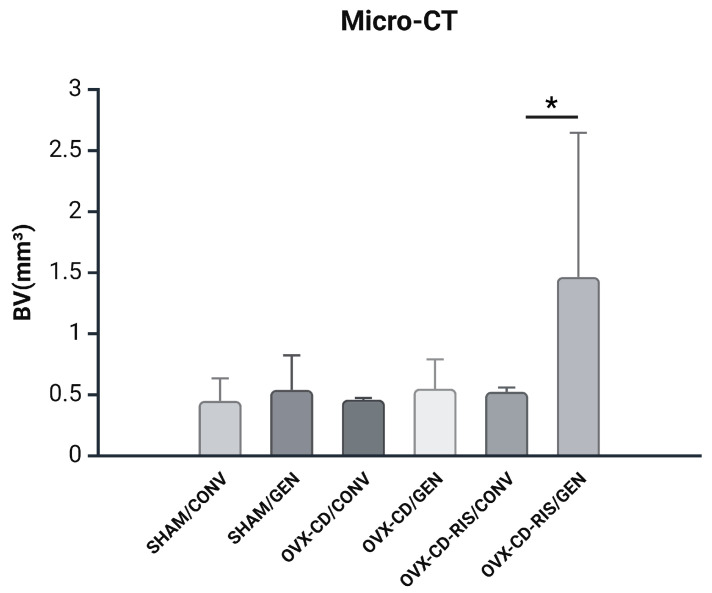
The Micro-CT values (BV) observed in experimental subgroups were analyzed using a two-way ANOVA, with the systemic condition of the animals and the implant surface type as the factors under evaluation. When significant differences were detected between the factors, the Tukey post-test was applied, with a significance level set at *p* < 0.05. An asterisk (*) indicates a statistically significant difference compared to the OVX-CD-RIS/GEN subgroup.

In BV, all subgroups with GEN implants tend to achieve higher bone volume values when compared to CONV implants, but there is no statistical difference (Figure 2). The OVX-CD-RIS/GEN subgroup achieved the highest result. Statistically significant differences were observed between OVX-CD-RIS/CONV vs. OVX-CD-RIS/GEN (*p* = 0.0039).

### 3.3. Epifluorescence

#### 3.3.1. Bone Dynamics

**Figure 3:**
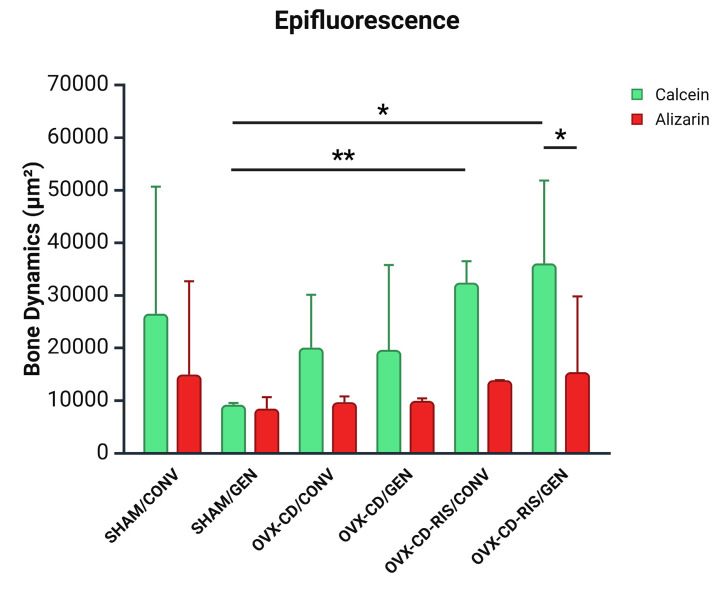
The values obtained in bone dynamics were submitted to the two-way ANOVA statistical test, with the fluorochromes of calcein in green and alizarin in red, and systemic conditions (systemic condition and different implants) tested as factors evaluated. If there were differences between the factors, the Tukey post-test was applied, with a significance level of (*p* < 0.05). An asterisk (*) indicates a statistically significant difference compared to the OVX-CD-RIS/GEN subgroup. Two asterisks (**) denote a statistically significant difference compared to the OVX-CD-RIS/CONV subgroup.

**Figure 4:**
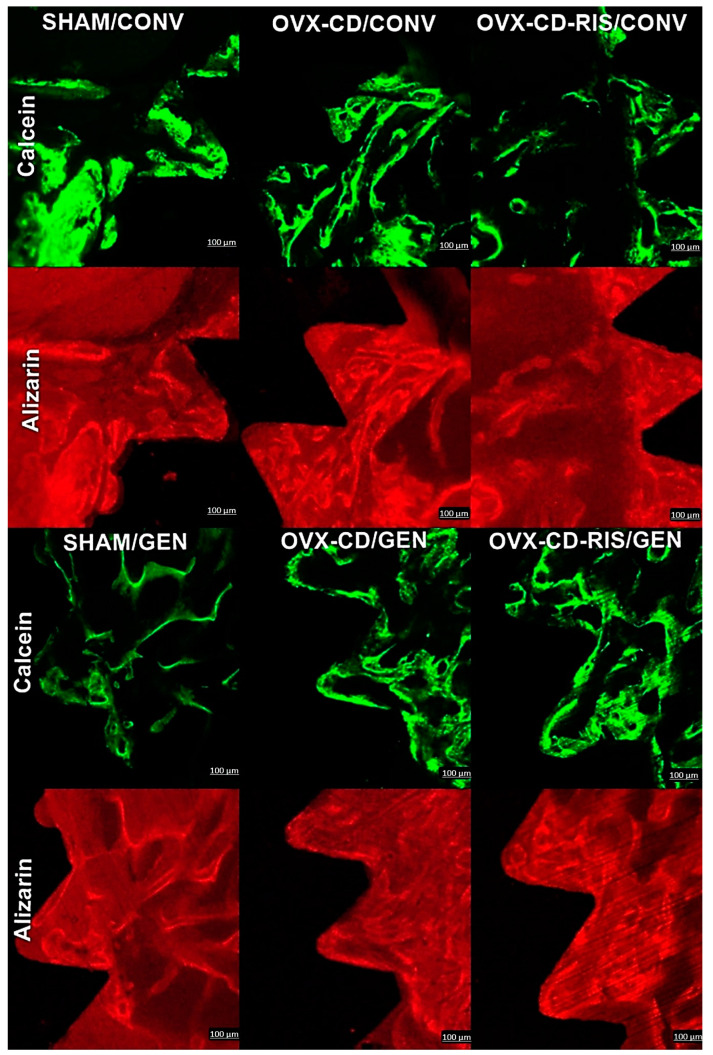
Confocal microscopy images were obtained in all subgroups, with the microscope filters adjusted to specifically detect calcein (green) and alizarin red (red) fluorescence. Calcein staining indicates an old bone, while alizarin red highlights areas of new bone. Images were analyzed using ImageJ software (Bethesda, MD, USA). Scale bar = 100 µm.

The peri-implant bone formed in the medullary bone was considered an area of interest when observing the precipitation of fluorochromes of calcein and alizarin. It was noticed that all subgroups showed greater precipitation of calcein than alizarin (Figure 3). Statistical differences were observed between both calcein values from SHAM GEN vs. OVX-CD-RIS/CONV (*p* = 0.0284) and SHAM GEN vs. OVX-CD-RIS GEN (*p* = 0.0070). In addition, a difference was noted between the calcein value from OVX-CD-RIS/GEN vs. the alizarin value from OVX-CD-RIS/GEN (*p* = 0.0316). Epifluorescence images on each fluorochrome are shown in Figure 4.

#### 3.3.2. Daily Mineral Apposition Rate (MAR)

**Figure 5:**
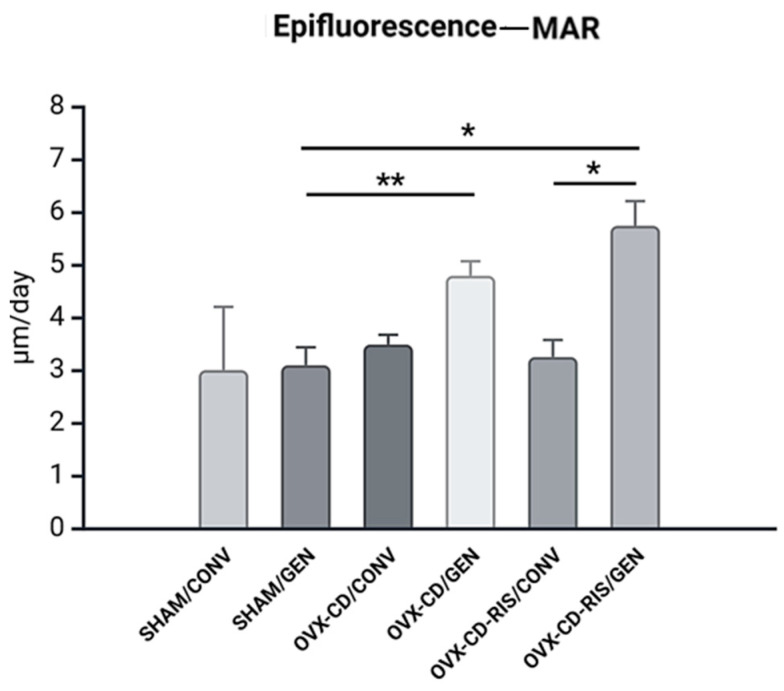
The MAR results were submitted to the two-way ANOVA statistical test, with the systemic condition of the animals and the surface of the implants tested as factors evaluated. If there were differences between the factors, the Tukey post-test was applied, with a significance level of (*p* < 0.05). An asterisk (*) indicates a statistically significant difference compared to the OVX-CD-RIS/GEN subgroup. Two asterisks (**) denote a statistically significant difference compared to the OVX-CD/GEN subgroup.

**Figure 6:**
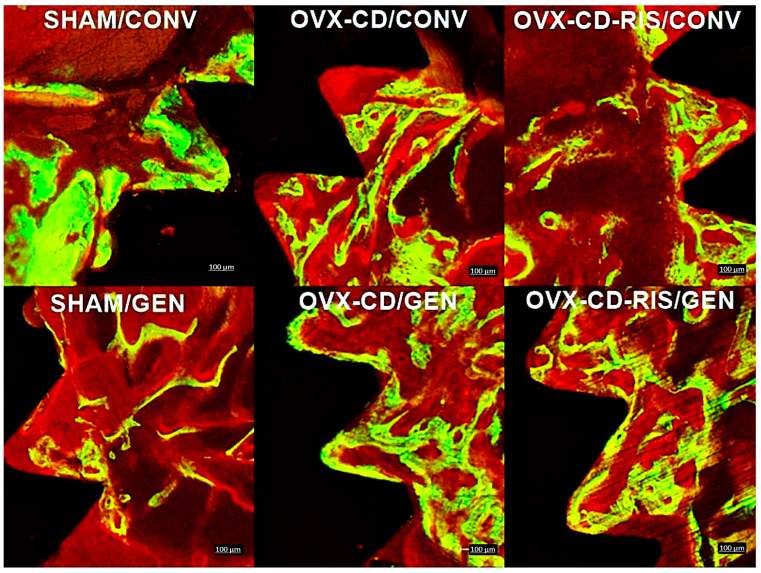
Images were captured using a confocal microscope for the overlapping of both fluorochromes using ImageJ software (Bethesda, MD, USA). Calcein green stain was used to visualize older bone, while alizarin red stained newly mineralized bone, highlighting areas of active calcification. Quantitative analysis included measuring the distance between the calcein and alizarin red staining ranges, which was then correlated to the time difference (in days) between injections, providing the daily mineral apposition rate (MAR). Scale bar = 100 µm.

For the MAR, higher values were observed in the subgroups treated with GEN, with the OVX-CD-RIS/GEN group having the highest value among the subgroups (Figure 5). Statistically significant differences were observed between the subgroups: SHAM/GEN vs. OVX-CD/GEN (*p* = 0.0152), SHAM/GEN vs. OVX-CD-RIS/GEN (*p* = 0.0016), and between OVX-CD-RIS/CONV vs. OVX-CD-RIS/GEN (*p* = 0.0022). The quantification of the MAR between the calcein and alizarin staining was performed by overlapping fluorochrome as shown in Figure 6.

### 3.4. Molecular Analysis (RT-qPCR)

**Figure 7:**
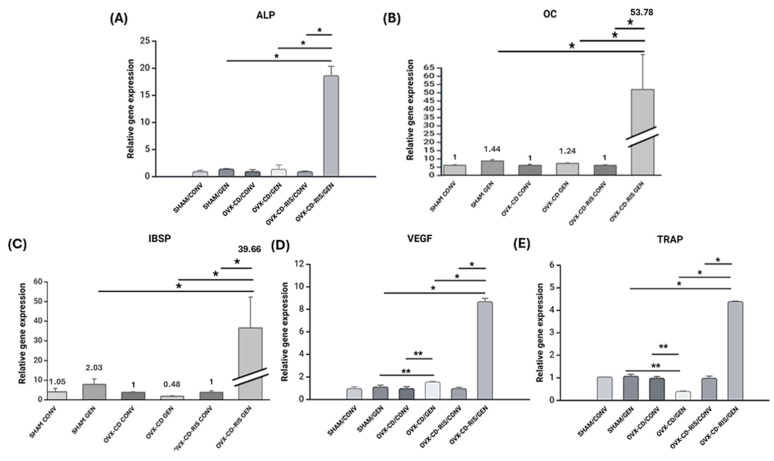
Bar graphs with mean and standard deviation from molecular analysis for each gene expression (**A**–**E**). The data were submitted to the two-way ANOVA statistical test, with the systemic condition and the surface of the implants tested as the factors evaluated. If there were differences between the factors, Tukey’s post-test was applied, with a significance level of (*p* < 0.05). An asterisk (*) indicates a statistically significant difference compared to the OVX-CD-RIS/GEN subgroup. Two asterisks (**) denote a statistically significant difference compared to the OVX-CD/GEN subgroup.

The relative gene expression of ALP (Figure 7A), OC (Figure 7B), IBSP (Figure 7C), VEGF (Figure 7D), and TRAP (Figure 7E) showed statistically significant differences (*p* < 0.05) between the following comparisons: SHAM/GEN vs. OVX-CD-RIS/GEN, OVX-CD/GEN vs. OVX-CD-RIS/GEN, and OVX-CD-RIS/CONV vs. OVX-CD-RIS/GEN. Additionally, significant differences (*p* < 0.05) were observed between the subgroups SHAM/GEN vs. OVX-CD/GEN and OVX-CD/CONV vs. OVX-CD/GEN for VEGF and TRAP.

### 3.5. Association Between Bone Microarchitecture and Osteogenesis Markers

The findings from the different analyses, including Micro-CT imaging and molecular assays, together provide a comprehensive understanding of the synergistic effects of systemic RIS and GEN-functionalized implants on peri-implant bone repair.

Micro-CT analysis revealed that the combination of systemic RIS and GEN-functionalized implants significantly enhanced peri-implant bone microarchitecture, with the OVX-CD-RIS/GEN subgroup showing a notable increase in BV. This increase in bone volume directly correlates with the elevated expression of osteogenic markers, such as OC and IBSP, observed in molecular assays. The higher expression of these markers indicates enhanced osteoblastic activity and bone mineralization, key processes involved in bone formation.

Moreover, the increased bone turnover observed in confocal microscopy (via higher calcein labeling and mineral apposition rates) further supports the findings from Micro-CT. The higher MAR in GEN-functionalized implants compared to conventional implants highlights the role of GEN in promoting osteogenesis, ensuring that bone formation and resorption are balanced even under estrogen-deficient conditions.

Thus, the integration of these analyses—Micro-CT for structural changes, molecular assays for markers of osteogenesis, and confocal microscopy for bone turnover—demonstrates how increased BV correlates with molecular markers of osteogenesis. The combined actions of RIS and GEN not only promote osteogenesis but also improve the overall bone quality around the implant, ensuring better osseointegration and supporting bone repair in compromised conditions like estrogen deficiency.

## 4. Discussion

The primary objective of this study was to assess the impact of combining systemic and local treatments on peri-implant repair in estrogen-deficient rats fed a CD. The main result determined a significant synergistic effect between systemic RIS and GEN-functionalized implants, particularly in the OVX-CD-RIS/GEN subgroup, which exhibited the highest removal torque values. This suggests that the combination therapy not only enhances mechanical stability but also improves bone quality around the implant [29]. In this sense, the micro-CT data revealed that the synergistic action of systemic RIS and local GEN significantly enhanced peri-implant bone microarchitecture, evidenced by increased BV in the OVX-CD-RIS/GEN subgroup. All these findings align with existing studies [30], highlighting the synergic effect of systemic therapy with local treatments in improving bone repair under compromised conditions by estrogen deficiency [27] and even associated with CD [17].

Other studies with molecular analyses indicated elevated expression levels of key extracellular matrix proteins crucial to bone dynamics [21,22]. In the findings, the expression of OC and IBSP was higher in the combined treatment in the OVX-CD-RIS/GEN subgroup. This is particularly relevant as the expression of these proteins is associated with stages of mineralization of extracellular matrix on bone [31]. Hence, these higher values reveal the potential of systemic RIS and local GEN to enhance bone maturation. Moreover, while RIS is known for its beneficial antiresorptive effects, it typically reduces vascularization as a risk to osteonecrosis of the jaw [13]. However, the incorporation of GEN-functionalized implants appeared to increase the expression of VEGF, a key factor in angiogenesis. This suggests that, while RIS exerts its antiresorptive properties, local GEN may play a vital role in ensuring vascularization. Importantly, vascularization is critical for supporting bone repair [32,33] and preventing medication-related osteonecrosis of the jaw [13]. Therefore, the combination of these therapies could work synergistically to improve peri-implant bone healing.

Confocal microscopy results further supported these findings, showing increased bone turnover in both the OVX-CD/GEN and OVX-CD-RIS/GEN subgroups. Specifically, the OVX-CD-RIS group exhibited the highest calcein labeling, regardless of implant GEN treatment, highlighting the antiresorptive action of systemic RIS. Additionally, the mineral apposition rate (MAR) was significantly higher in GEN-functionalized implants compared to conventional implants in the OVX-CD-RIS group. This suggests that the combination of systemic RIS and local GEN may help maintain a balanced bone turnover between resorption and formation, particularly in the context of estrogen deficiency and CD. Lastly, the MAR values from epifluorescence analysis were notably higher in both the OVX-CD/GEN and OVX-CD-RIS/GEN subgroups compared to the SHAM/GEN subgroup, emphasizing the local role of GEN in preventing bone resorption mainly under compromised conditions.

Previous studies provide strong evidence that aligns with the findings of the present study. The antiresorptive assets of GEN on bone are demonstrated through the modulation of osteoprotegerin and RANKL [30,31] and increasing OC expression [34]. GEN shares structural similarities with estrogen [35] and consequently promotes osteoblast recruitment via activating estrogen receptors [30,33]. The ability of GEN to enhance peri-implant bone repair has also been verified in earlier research [36,37], but that study did not combine any synergistic treatments. In this prior study, implants loaded with GEN resulted in increased peri-implant bone repair in rats with estrogen deficiency [34]. On the other hand, it is essential to underline the impact of RIS in the treatment of osteoporosis [38]. In this context, a prior systematic review indicates a lower risk of osteonecrosis of the jaw since it is intake via oral RIS [39]. Importantly, our study observed no signs of osteonecrosis in any case. The effect of GEN in promoting angiogenesis can avoid it; however, it also did not occur in the OVX-CD-RIS/CONV subgroup.

The functionality of implants coated with GEN appears to be particularly beneficial when combined with systemic RIS, as it not only improves osseointegration but also promotes a healthier bone microenvironment, especially in bone metabolism and turnover. The clinical relevance of this research should be emphasized. These findings suggest a promising strategy for patients undergoing osteoporosis treatment with bisphosphonates, emphasizing the need for more in-depth studies to elucidate the local effects of GEN on osseointegration and overall bone repair. Lastly, the synergistic effects of RIS and GEN present a compelling case for combined therapy in improving peri-implant outcomes in compromised bone conditions. Among the various limitations of this preclinical study, the absence of analysis of decalcified sections and the single dose of the drugs studied must be included. Thus, future research should focus on further characterizing these interactions and optimizing treatment protocols to enhance bone healing in clinical settings.

Based on the discussion, it can be speculated that GEN’s effects are primarily both osteogenic and angiogenic. The combination of systemic RIS and GEN-functionalized implants enhances bone repair in estrogen-deficient conditions, where both osteogenesis and vascularization are critical. GEN’s osteogenic effects are supported by its ability to stimulate the expression of OC and osteoblast-related proteins such as IBSP, suggesting its role in promoting bone formation. Additionally, GEN enhances vascularization through the upregulation of VEGF, a key angiogenic factor, which is vital for supporting bone repair. While RIS primarily exerts anti-resorptive effects by reducing bone turnover and improving bone density, the inclusion of GEN-functionalized implants ensures proper bone maturation and vascular support. Thus, GEN’s dual action, promoting both osteogenesis and angiogenesis, complements the anti-resorptive properties of RIS, contributing to more effective peri-implant healing and better osseointegration in compromised bone conditions.

Lastly, our study demonstrates that combining systemic RIS and GEN-functionalized implants shows promise for improving peri-implant outcomes in patients with compromised bone conditions, such as osteoporosis and metabolic syndrome. This dual approach may enhance both bone density and vascularization, addressing concerns about implant failure and osteonecrosis of the jaw associated with bisphosphonates. However, translating these findings to human clinical settings may involve challenges, such as differences in human and rat metabolism and the long-term effects of RIS and GEN on bone healing. While the combination therapy appears beneficial, clinical trials are needed to optimize treatment protocols, assess potential adverse effects, and explore the long-term impact on implant stability and bone repair in diverse patient populations. These trials should consider dosage variations, patient comorbidities, and longer-term outcomes to ensure the safety and efficacy of the combined therapy in human applications.

## 5. Conclusions

This study demonstrates that the combination of systemic RIS and local GEN-functionalized implants significantly enhances peri-implant repair in estrogen deficiency rats on a cafeteria diet. Future research should focus on exploring variable dosages and treatment protocols to optimize this combined therapy, with the goal of enhancing peri-implant outcomes and facilitating its translation into clinical practice for postmenopausal women with metabolic syndrome.

## 6. Patents

BR 10 2021 019134 1 process—“Funcionalização da superfície de implantes com genisteina através da técnica de layer by layer como estratégia para a melhora do processo de preparo periimplantar em ratas” (870210088311 petition).

## Figures and Tables

**Table 1:** Experimental study subgroup overview.

Groups	Systemic Condition	Subgroups	Local Treatment
SHAM	Rats with estrogen	SHAM/CONV	Received CONV implants
	Rats with estrogen	SHAM/GEN	Received implants functionalized with GEN
OVX-CD	Rats with estrogen deficiency and CD	OVX-CD/CONV	Received CONV implants
	Rats with estrogen deficiency and CD	OVX-CD/GEN	Received implants functionalized with GEN
OVX-CD-RIS	Rats with estrogen deficiency, CD, and systemic RIS	OVX-CD-RIS/CONV	Received CONV implants
	Rats with estrogen deficiency, CD, and systemic RIS	OVX-CD-RIS/GEN	Received implants functionalized with GEN

**Table 2:** Taqman probes for real-time PCR.

Gene	Gene Reference	Forward Primer, 5′ → 3′	Reverse Primer, 5′ → 3′
ALP	NM_013059.1	GAGGAACGGATCTCGGGGTA	ATGAGTTGGTAAGGCAGGGTC
OC	NM_013414.1	CTCTGAGTCTGACAAAGCCTTCAT	GTAGCGCCGGAGTCTATTCA
IBSP	NM_012587.2	GTACCGGCCACGCTACTTTC	ATCTCCAGCCTTCTTGGGTAGC
VEGF	NM_057149.2	GCACTCCTGGTGTTCTTGGA	TTTGGTCCCAGGCAAACTGT
TRAP	NM_019144.2	TCTCAGCGATCACCGCTTC	ATCCATGCTGGAGGAAGCAC
ß-actin	NM_031144.3	CCACCATGTACCCAGGCATT	CCTAGAAGCATTTGCGGTGC

## Data Availability

The original contributions presented in this study are included in the article. Further inquiries can be directed to the corresponding authors.
